# Breaking Open the Black Box: Isolating the Most Potent Features of a Web and Mobile Phone-Based Intervention for Depression, Anxiety, and Stress

**DOI:** 10.2196/mental.3573

**Published:** 2015-03-04

**Authors:** Alexis E Whitton, Judith Proudfoot, Janine Clarke, Mary-Rose Birch, Gordon Parker, Vijaya Manicavasagar, Dusan Hadzi-Pavlovic

**Affiliations:** ^1^The Black Dog InstituteUniversity of New South WalesSydneyAustralia; ^2^School of PsychiatryFaculty of MedicineUniversity of New South WalesSydneyAustralia

**Keywords:** eHealth, depression, anxiety, stress, psychological stress, self-help, Web-based, mental health

## Abstract

**Background:**

Internet-delivered mental health (eMental Health) interventions produce treatment effects similar to those observed in face-to-face treatment. However, there is a large degree of variation in treatment effects observed from program to program, and eMental Health interventions remain somewhat of a black box in terms of the mechanisms by which they exert their therapeutic benefit. Trials of eMental Health interventions typically use large sample sizes and therefore provide an ideal context within which to systematically investigate the therapeutic benefit of specific program features. Furthermore, the growth and impact of mobile phone technology within eMental Health interventions provides an opportunity to examine associations between symptom improvement and the use of program features delivered across computer and mobile phone platforms.

**Objective:**

The objective of this study was to identify the patterns of program usage associated with treatment outcome in a randomized controlled trial (RCT) of a fully automated, mobile phone- and Web-based self-help program, “myCompass”, for individuals with mild-to-moderate symptoms of depression, anxiety, and/or stress. The core features of the program include interactive psychotherapy modules, a symptom tracking feature, short motivational messages, symptom tracking reminders, and a diary, with many of these features accessible via both computer and mobile phone.

**Methods:**

Patterns of program usage were recorded for 231 participants with mild-to-moderate depression, anxiety, and/or stress, and who were randomly allocated to receive access to myCompass for seven weeks during the RCT. Depression, anxiety, stress, and functional impairment were examined at baseline and at eight weeks.

**Results:**

Log data indicated that the most commonly used components were the short motivational messages (used by 68.4%, 158/231 of participants) and the symptom tracking feature (used by 61.5%, 142/231 of participants). Further, after controlling for baseline symptom severity, increased use of these alert features was associated with significant improvements in anxiety and functional impairment. Associations between use of symptom tracking reminders and improved treatment outcome remained significant after controlling for frequency of symptom tracking. Although correlations were not statistically significant, reminders received via SMS (ie, text message) were more strongly associated with symptom reduction than were reminders received via email.

**Conclusions:**

These findings indicate that alerts may be an especially potent component of eMental Health interventions, both via their association with enhanced program usage, as well as independently. Although there was evidence of a stronger association between symptom improvement and use of alerts via the mobile phone platform, the degree of overlap between use of email and SMS alerts may have precluded identification of alert delivery modalities that were most strongly associated with symptom reduction. Future research using random assignment to computer and mobile delivery is needed to fully determine the most ideal platform for delivery of this and other features of online interventions.

**Trial Registration:**

Australian New Zealand Clinical Trials Registry (ACTRN): 12610000625077; http://www.anzctr.org.au/TrialSearch.aspx? (Archived by WebCite http://www.webcitation.org/6WPqHK0mQ).

## Introduction

### eMental Health Interventions

When used correctly, Internet-delivered mental health (eMental Health) interventions produce treatment effects similar to those observed in face-to-face treatment [1-4]. However, treatment effects observed vary distinctly across programs [5], and eMental Health interventions still remain somewhat of a black box in terms of our understanding of the mechanisms by which they exert their therapeutic benefit.

Existing frameworks posit a range of features that are most likely to influence program usage and treatment outcome [6], and recent reviews have attempted to isolate the features that mediate intervention outcomes [7]. However, there remains a large degree of variability in the number and types of features included in eMental Health interventions. As these interventions become increasingly more sophisticated, multifaceted, and user-driven, the heterogeneity in patterns of usage, and subsequently, the number of treatment components, also increases. This makes it difficult to identify those features of greatest therapeutic benefit.

### Associations Between Patterns of Program Usage and Symptom Reduction

Within trials of eMental Health interventions, examining user log data provide an ideal means of identifying the patterns of program usage that are associated with the greatest therapeutic gain. As such, research has recently turned to focus on how specific patterns of program usage impact on treatment outcome. For example, in a trial of a Web-based intervention for diabetes self-management, use of a self-monitoring feature was associated with improvements in healthy eating, reductions in dietary fat, and increased exercise, but not with medication compliance [8]. Similarly, in a study examining the relationship between use of features of a personally controlled health management record and help-seeking behaviors, Lau et al [9] found that increased use of a diary feature, an Internet poll feature, and an Internet appointment booking feature, correlated with increased help-seeking for emotional well-being as well as overall health service utilization. Studies have also found that specific site usage statistics predict treatment outcome, such as the number of program log-ins [10], the number of psychotherapy module pages accessed [11], the number of activities completed per log-in [12], and use of reminder emails [13]. These finer-grained analyses of the relationship between program use and treatment outcome represent an important first step in developing an understanding of what specific program features impact most on treatment outcome. However, further studies of multifaceted programs are needed if we are to identify the most potent features of eMental Health interventions.

### Incorporation of Mobile Phone Technology

The growth and impact of mobile phone-based interventions provides an opportunity to examine the therapeutic benefits of adding a mobile phone platform to deliver program functions. A particular advantage of mobile phone technology is its capacity to facilitate ecological momentary assessment (EMA); that is, self-monitoring of symptoms and behaviors in real-time and in real-world contexts [14,15]. Self-monitoring of symptoms and behaviors in the context in which they occur helps to promote improved self-awareness of the factors that contribute to depression, stress, and anxiety. Therefore, it is likely that accessing features of an Internet intervention via a mobile phone platform may enhance treatment outcomes. However, no studies to date have examined the therapeutic gains associated with accessing program features via mobile technology.

### Current Research

The current study examined associations between patterns of program usage and improvements in symptoms of depression, anxiety, stress, as well as functional impairment, in a randomized controlled trial (RCT) of a Web and mobile phone intervention for individuals with mild-to-moderate mental health symptoms. The current study also examined associations between symptom reduction and use of features across both computer and mobile phone platforms, thereby providing novel insights into the potential benefits of incorporating mobile technology into Web-based interventions.

Known as “myCompass”, the program under current consideration is a stand-alone, fully automated self-help program that contains a variety of therapeutic features including interactive psychotherapy modules, self-monitoring, a diary, automated reminders, and motivational messages. The outcomes from the myCompass RCT are described elsewhere [16]. During the RCT, participants who were assigned to receive the myCompass intervention were able to engage with the program in an unrestricted manner, providing us with an ideal opportunity to determine what features were used most often and what were most closely associated with improvements in symptoms and functional impairment. Furthermore, the myCompass program is among the first to incorporate the use of mobile technology. Users could choose to access some of the program features on their computer or mobile phone, and could also choose to receive alerts via email or short message service (SMS; ie, text message). This also provided us with the opportunity to examine whether the addition of a mobile phone platform was associated with increased use of other program features, as well as improvements in symptoms and functional impairment.

The aims of this study were to describe the site usage patterns of participants who received access to the myCompass program for seven weeks during the RCT, and to identify the program features associated with enhanced program engagement and reductions in depression, anxiety, stress, and functional impairment.

## Methods

### Participants

In the RCT, participants were randomly allocated to the myCompass intervention, to an attention control condition, or to a waitlist control condition, for seven weeks. The RCT took place between October 2011 and June 2012, during which time usage data were recorded. The usage data presented here are derived from all participants who were randomly allocated to the myCompass intervention condition (n=231).

### The myCompass Program

The myCompass program is a fully automated, interactive, stand-alone mobile phone and Internet-delivered intervention designed for the treatment of mild-to-moderate symptoms of depression, anxiety, and stress. Users can access the program through any Internet-enabled device, including smart phones, tablets, laptops, and desktop computers. The program comprises four key features that users can access and engage with in a manner they choose.

### Modules

The program contains 12 modules that are based on principles of cognitive behavior therapy, interpersonal psychotherapy, and problem solving approaches. These modules are: (1) Managing Stress and Overload, (2) Communicating Clearly, (3) Problem Solving, (4) Tackling Unhelpful Thinking, (5) Fear and Anxiety, (6) Happiness, (7) Sleep, (8) Relaxation, (9) Taking Charge of Worry, (10) Increasing Pleasurable Activities, (11) Smart Goals, and (12) Managing Loss. Modules are accessed via the Web only on computers (due to the size of the content, they were unavailable on the mobile phone), each module is approximately 10 Web pages in length and is divided into three sections that take approximately 5-10 minutes to complete, with a homework exercise at the end. Users are encouraged to complete one module session per week (with most modules containing 2-3 sessions), with the aim of completing 2 full modules over the course of the intervention. Participants are also encouraged to complete the homework tasks in between sessions. Module sessions are delivered in a tunnelled manner, such that it is necessary to complete a section and enter homework data before moving on to the next section.

### Symptom Tracking

A number of predefined tracking dimensions are available for users to select from: (1) anxiety, (2) depression, (3) irritability, (4) restlessness, (5) stress, (6) worry, (7) alcohol, (8) diet, (9) exercise, (10) medication, (11) sleep, (12) smoking, (13) confidence, (14) concentration, (15) energy, and (16) motivation. In addition, users can define their own dimension to track. A maximum of three dimensions can be tracked at one time. Users can choose to track daily or weekly and to receive tracking reminders via email or SMS. Each time a user enters tracking information, they are also prompted to enter situational information, such as where they are (eg, work, home, commuting), whom they are with (eg, colleagues, friends, alone), and what they doing (eg, working, studying, socializing) in order to help users identify potential triggers to their mood symptoms.

### “Snippets”

“Snippets” are brief mental health tips, facts, and motivational messages that a user can elect to receive via SMS or email throughout the intervention. Users can choose the frequency with which they receive snippets, and can turn the feature on or off whenever they choose.

### Diary

myCompass also contains a diary feature in which users can make notes about insights they have gained or information they have learned while engaging with the program. For example, users may use the diary to make notes about situational factors that appear to be linked with their mood symptoms on the basis of their symptom tracking data.

### Primary Outcome

The Depression, Anxiety, and Stress Scale (DASS-21) [17] is a 21-item self-report scale that was used to measure symptoms of depression, anxiety, and stress. Respondents rate how much they have been bothered by symptoms over the past week on a 4-point Likert scale from 0 (*Did not apply to me at all*) to 3 (*Applied to me very much/most of the time*). The DASS-21 has adequate internal consistency and test-retest reliability [17], and has sound psychometric properties when administered in an Internet format [18]. Total scores range from 0 to 126 and subscale scores range from 0 to 42, with higher scores indicative of more severe symptomatology.

### Secondary Outcome

The Work and Social Adjustment Scale (WSAS) [19] is a 5-item self-report scale that was used to assess the degree to which mental health problems interfere with daily functioning. Respondents indicate the degree to which their mental health problems lead to functional impairment in the domains of work, social leisure activities, private leisure activities, home management, and personal relationships, on a 9-point Likert scale from 0 (*Not at all*) to 8 (*Very severely*). The WSAS has been shown to have sound psychometric properties when administered on the Internet [20], and higher scores indicate greater levels of functional impairment.

Demographic information was also collected as part of the baseline questionnaire, and included age, gender, highest level of education attained, and employment status. Frequency with which the participant used mobile phones and computers provided a proxy measure of technology acceptance.

Treatment outcome measures were administered at baseline, 8 weeks after baseline, and at 5 months after baseline. In this paper, we report the relationship between patterns of usage and treatment outcomes specifically during the intervention period (ie, baseline to 7 weeks).

### Usage Outcomes

Data for the following usage variables were obtained: log-ins, modules, symptom tracking, snippets, and diary.

Log-ins was the total number of times a user logged in to the myCompass program.

Modules were the number of modules a user started, the number they completed, and which modules they completed.

Symptom tracking was the number of times a user entered symptom tracking data, the number of tracking reminders a user received (by SMS and email), and the number of times a user tracked a particular symptom. A tracking latency variable was also calculated to determine how long, on average, it took for users to respond to tracking reminders by entering tracking data. This was calculated as the average length of time (in hours) between when a tracking reminder was received and the next instance of entering tracking data.

“Snippets” were the number of “snippets” a user received via SMS and via email.

Diary was the number of diary entries a user made.

### Analysis Strategy

First, the sample was divided into two groups, those who used and those who did not use a specific feature. Chi-square tests (for categorical variables) and separate univariate Analyses of Variance (for continuous demographic variables) were then used to test for differences between the two groups on demographics and baseline scores on the DASS subscales and WSAS. Second, for those who used a specific feature, partial correlations were carried out to determine whether frequency of feature use was correlated with post intervention DASS or WSAS scores, after controlling for baseline scores on these measures.

## Results

### Participants

Of the 720 participants who screened eligible to participate in the myCompass RCT, 242 were randomly allocated to receive the myCompass program for seven weeks. There were eleven participants in this group that withdrew from the RCT, leaving a final sample of 231 for the current analysis. The sample was predominantly female (160/231, 69.3%), university educated (135/231, 58.4%), employed (196/231, 84.8%), married or de facto (133/231, 57.6%), and the mean age was 38.8 years. The majority reported using a computer (225/231, 97.4%) and a mobile phone (223/231, 96.5%) daily.

### Patterns of Site Usage

Google Analytics was used to extract site usage data for those in the myCompass group over the course of the RCT, spanning from October 4, 2011 to June 25, 2012. Google Analytics defines visits as the number of times visitors have been to the website. If a visitor is inactive for 30 minutes or more, any future activity is attributed to a new session. Visitors who leave the site and return within the same 30-minute period are counted as part of the same session. During the RCT period, there were a total of 4724 visits to the site, 1182/4724 (25.02%) of which were unique (ie, first time) visits. Across the 266 days, the site recorded 58,296 page views, with an average of 12.34 page views per visit and an average visiting time of 10 minutes and 37 seconds. The bounce rate, which reflects the proportion of visitors who enter the site and leave (“bounce”) without viewing other pages within the site, was 19.37%.

**Figure 1:**
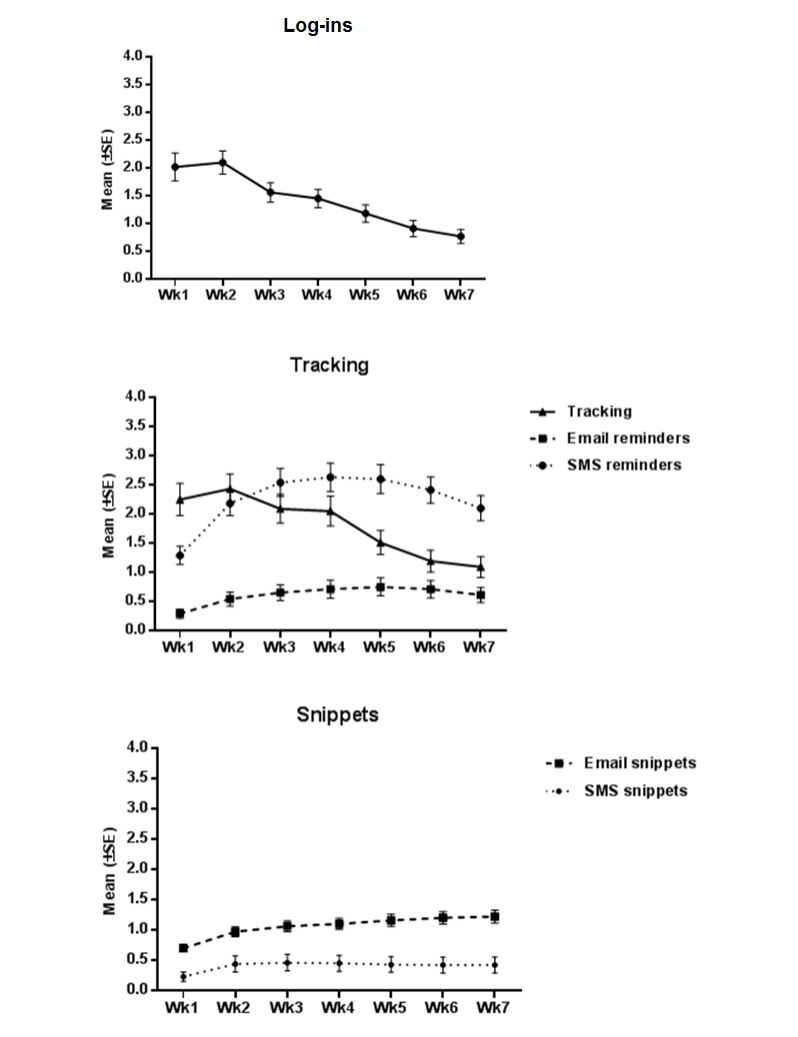
Use of program features across the intervention. SMS = short message service; Week = Wk.

Participants were given access to the myCompass program for seven weeks. They were encouraged to track three symptom dimensions and complete two modules of their choice during the intervention period. A little under a third (n=66/231, 28.6%) of the participants never logged in to the program. Those who did use myCompass logged in a total of 2463 times, with the mean number of log-ins per user being 14.9 (SD 16.5), ranging from 1 to 105. The most commonly used feature of the program was the “snippets” feature, used by 158/231 (68.4%) participants. This was followed by the symptom tracking feature, used by 142/231 (61.5%) participants, and 118/231 (51.1%) also used the optional tracking reminder feature. There were 118/231 (51.1%) participants who also used the modules, 57 of whom completed at least one module. The mean number of modules started was 1.2 (SD 1.6) and the mean number completed was 0.6 (SD 1.3). The least commonly used feature was the diary, used by 61/231 (26.4%) participants. Key usage statistics of those who used the program (n=165) are shown in Tables 1-3. Figure 1 shows the use of specific program features over the seven-week intervention period.

**Table 1:** Module usage statistics (n=165).

Completion of specific modules	Started (n=118^a^)		Completed (n=57^b^)	
	n	% sample	n	% sample
Managing stress and overload	63	38.2	33	20.0
Communicating clearly	43	26.1	16	9.7
Problem solving	34	20.6	17	10.3
Tackling unhelpful thinking	26	15.8	13	7.9
Fear and anxiety	25	15.2	9	5.5
Happiness	22	13.3	11	6.7
Sleep	13	7.9	5	3.0
Relaxation	12	7.3	2	1.2
Taking charge of worry	10	6.1	7	4.2
Increasing pleasurable activities	9	5.5	5	3.0
Smart goals	6	3.6	6	3.6
Loss	5	3.0	3	1.8

^a^ Indicates the number of users who started at least one module

^b^ Indicates the number of users who completed at least one module. Percentages refer to the portion of users who started/completed a module with respect to the total sample of users who logged on at least once, n=165.

**Table 2:** Symptom tracking usage statistics (n=165).

Symptom tracking		Median	Mean	SD
Instances of tracking		31	49.9	54.2
**Reminders**				
	Number of email reminders received	0	6.7	15.9
	Number of SMS reminders received	24	25.4	26.2
**Frequency of tracking specific dimensions**				
	Stress	0	6.2	16.6
	Anxiety	0	5.9	14.9
	Worry	0	5.1	14.0
	Depression	0	4.9	13.4
	Motivation	0	4.9	11.9
	Energy	0	4.3	12.7
	Irritability	0	3.6	10.4
	Confidence	0	3.1	10.5
	Restlessness	0	2.6	10.7
	Sleeping	0	2.4	6.1
	Exercise	0	1.7	4.9
	Concentration	0	0.7	4.4
	Diet	0	0.6	2.5
	Alcohol	0	0.5	2.8
	Smoking	0	0.1	0.5
	Medication	0	0.0	0.1
Latency (hours)		55	9.5	34.9

**Table 3:** Snippets usage statistics (n=165).

Snippets		Median	Mean	SD
**Total**				
	Number sent by email	7	7.2	6.9
	Number sent by SMS	0	2.8	10.3
**Quick tips**				
	By email	1	1.7	4.2
	By SMS	0	1.1	4.9
**Fast facts**				
	By email	0	0.2	1.0
	By SMS	0	0.1	0.5
**Motivational messages**				
	By email	5	5.2	2.9
	By SMS	0	1.6	6.3

### Baseline Predictors of Site Usage

Of the 231 individuals who received access to the myCompass program for 7 weeks during the RCT, 165 individuals logged in at least once. Reasons for nonusage included lack of time, poor motivation, and having intermittent access to the Internet or a computer. There were no significant differences between those who did and did not log in in terms of baseline demographics or clinical symptoms, however, for those who did use the program, log-in frequency (*r*=.19, *P*=.02) and total instances of symptom tracking (*r*=.18, *P*=.03) increased with participant age. Baseline demographic and symptom characteristics were not associated with frequency of starting or completing modules. Females chose to receive more “snippets” than males *t*_155_ = 2.15, *P*=.03, as did those with lower functional impairment (WSAS score; *r*=-.21, *P*=.04), and higher baseline anxiety was associated with a greater number of diary entries (*r*=.27, *P*=.04).

### Features Associated With Treatment Outcome

#### Modules

Neither the number of modules started nor the number of modules completed was significantly associated with reductions in depression, anxiety, stress, and functional impairment, after controlling for baseline symptom severity.

#### Symptom Tracking

Similarly, use of the symptom tracking feature was not associated with reductions in depression, anxiety, stress, or functional impairment, nor was the frequency of symptom tracking. There was also no correlation between symptom tracking latency and symptom improvement.

#### Symptom Tracking Reminders

After controlling for baseline severity, increased use of the symptom tracking reminders was associated with decreased functional impairment at post intervention (*r*=-.28, *P*=.01), however, there was no association between reminder use and depression, anxiety, or stress. Of those who used tracking reminders, 29 received at least one reminder via email, 97 received at least one reminder via SMS, and 8 subjects received reminders via both modalities. After controlling for baseline severity, frequency of tracking reminders received via email (*r*=-.27, *P*=.28), and via SMS (*r*=-.12, *P*=.37) were both associated with decreased functional impairment at post intervention, although neither correlation was significant when examined individually.

Bivariate correlations were conducted to determine whether reminders were associated with increased program use. The total number of tracking reminders was positively correlated with the number of program log-ins, as well as the frequency of symptom tracking (Table 4). A greater number of tracking reminders received via email were also associated with increased use and completion of the modules, as well as use of the diary. On each measure, reminders received via email were more strongly correlated with site usage than were reminders received via SMS. Interestingly, the correlation between tracking reminders and functional impairment remained significant even after controlling for frequency of symptom tracking (*r*=-.28, *P*=.02), indicating that reminders were associated with improvements in functional impairment independently of any associated increase in symptom tracking.

**Table 4:** Correlations between usage variables.

Reminders	Total tracking	Tracking latency	Program log-ins	Modules started	Modules completed	Diary entries
	*r* (*P* value)	*r* (*P* value)	*r* (*P* value)	*r* (*P* value)	*r* (*P* value)	*r* (*P* value)
Total	.21 (.02)	-.14 (.15)	.23 (.01)	-.07 (.50)	-.10 (.49)	.11 (.22)
SMS	.03 (.75)	-.12 (.25)	.05 (.65)	-.22 (.05)	-.22 (.20)	.05 (.62)
Email	.28 (.15)	.05 (.82)	.31 (.10)	.40 (<.001)	.40 (.003)	.43 (<.001)

#### “Snippets”

After controlling for baseline symptom severity, a greater number of snippets received were associated with reduced anxiety at post intervention (*r*=-.20, *P*=.04). Increased use of snippets was also associated with nonsignificant reductions in post intervention depression (*r*=-.09, *P*=.39), stress (*r*=-.17, *P*=.09), and functional impairment (*r*=-.27, *P*=.40). Of those who used snippets, 153 received at least one via email and 15 received at least one via SMS. Partial correlations between the number of email snippets received and post intervention scores were nonsignificant, as were partial correlations involving the number of SMS snippets. The lack of difference between the email and SMS delivery modes is possibly due to a high degree of overlap between the two, as 10 of the 15 users who received snippets via SMS also received snippets via email.

#### Diary

Use of the diary feature was not correlated with treatment outcomes.

## Discussion

### Aims of the Study

The aims of this study were: (1) to use log data to identify patterns of program use in an RCT of myCompass, a tailored, Web and mobile phone-based intervention for depression, anxiety, and stress; and (2) to determine whether use of certain program features was associated with enhanced program engagement and therapeutic gain. Overall, the results showed that the alert features of the myCompass program were the most commonly used, were associated with increased rates of program engagement, and were associated with the greatest therapeutic gains.

### Patterns of Program Use

The average number of log-ins per user over the course of the intervention was 14.9, which is somewhat higher than that reported in other eMental Health interventions (eg, a mean of 6.55 log-ins for individuals in the highest quartile of usage in an Internet intervention for depression) [10]. The bounce rate of 19.37% was also substantially lower than that observed in other interventions (eg, 32.9% in an intervention promoting sexual health, and 56.3% in an intervention promoting heart-healthy behaviors) [21,22], indicating that the home page was effective at engaging users. Furthermore, Google Analytics data revealed an average site visit duration of 10 minutes and 37 seconds, which is in line with that shown in other trials of Internet-delivered interventions for anxiety and depression that report average site visit durations of between 7 and 13 minutes [23,24], indicating a high level of engagement with the myCompass website. However, as is common in nontherapist-assisted eMental Health interventions [22,25,26], frequency of program use decreased over the course of the intervention, emphasizing the need to identify program features that promote more regular program engagement.

In the RCT, participants were encouraged to complete at least one myCompass module session per week (totalling 2 full modules over the course of the intervention). Log data revealed that module completion rates were lower than expected, with a mean module completion rate of 0.6 modules. This may be partly due to the fact that modules could not be accessed via the mobile phone due to screen size, and reformatting the modules for compatibility with a mobile phone platform is one obvious means of addressing this issue. Low module completion rates may also have arisen because the module sessions were delivered in a tunnelled fashion, whereby subjects were required to complete the module sessions in a predetermined sequence. Although research suggests that a tunnelled format may facilitate greater program engagement (eg, greater number of website pages visited, greater time spent on the website, and greater knowledge gained from the website) [27], subjects were often required to complete the between-session homework tasks before being able to progress to the next session within a module. Making homework tasks optional might be one way to increase module completion, by allowing greater flexibility in the speed with which users proceed through the modules.

The most commonly used feature of myCompass was the “snippets” function. “Snippets” were short motivational messages, quotes or facts, which were sent by email or SMS at a time and frequency chosen by the user. The appeal of the “snippets” may lie in the fact that they are supportive, normalizing, and instill hope, yet demand little of the user in terms of behavior change, attention, or time. The second most commonly used feature was the symptom tracking function. Among the most common symptom dimensions tracked were stress, worry, anxiety, motivation, and depression, indicating that users chose to track symptom dimensions most closely tied to the primary goal of the study, which was to reduce symptoms of depression, anxiety, and stress.

A substantial proportion of participants who used the symptom tracking feature also chose to receive tracking reminders. As expected, increased frequency of tracking reminders was associated with increased frequency of symptom tracking. This finding aligns with those of previous research showing that use of reminders enhances engagement with eMental Health interventions [28]. In particular, one study showed that users who received email reminders made 1.2 times as many visits, viewed 1.58 times as many pages, and spent 1.51 times as many minutes in an Internet intervention for smoking cessation [13], compared to users who did not receive email reminders. The majority of individuals who chose to receive reminders elected to receive them via SMS. Given that mobile phones are generally carried on the person, this finding may represent the perception that reminders are likely to be received in a more timely fashion if delivered to the mobile phone. It also suggests that program utilization may be enhanced (particularly use of features that involve alerts) via incorporating a mobile phone platform into future eMental Health interventions.

### Relationship Between Feature Use and Treatment Outcome

After controlling for baseline symptom severity, participants who chose to use the alert-based features of myCompass, such as the reminders and “snippets”, showed significantly greater reductions in post intervention symptoms and functional impairment compared to those who did not use these functions. In particular, there appeared to be a consistent relationship between alerts received via email and reductions in post intervention anxiety and functional impairment. In one respect, this is unsurprising, as self-monitoring is one of the most important components of psychotherapy for anxiety and other mental illnesses [29], and is a key step in helping individuals to change unhelpful cognitions, beliefs, and behaviors [30]. Furthermore, the greater association between email reminders and treatment outcome may be because users who chose to receive alerts via email spend more time on their computer, and as a result, could more easily access the program features that were only available via the computer platform (such as the modules). However, the relationship between email reminders and treatment outcome remained significant even when controlling for frequency of program use and symptom tracking. Although the alert functions of myCompass were primarily intended to enhance program engagement (which they ostensibly did), these data suggest that alerts may also impact treatment outcome independently of their effects on program engagement.

This finding indicates that alerts may serve multiple therapeutic functions and may therefore be an especially potent feature in eMental Health interventions. First, in terms of program usage, alerts act as prompts that enhance program engagement, and so may help to combat the high rates of nonusage attrition common to many Internet interventions [31,32]. Alerts may also be useful for encouraging more ongoing program engagement in users who show a tendency to prematurely disengage from the program once they have begun to achieve symptom remission (“e-attainers”). Second, in terms of treatment outcome, alerts may independently produce therapeutic effects via nonspecific therapeutic processes. Nonspecific variables pertinent to the treatment context, such as encouragement, empathy, and hopefulness of improvement can produce powerful treatment effects in their own right, for a review see [33]. Therefore, receiving regular reminders, motivational messages, and tips may lead to reductions in symptomatology and functional impairment because they regularly and consistently cue the expectation of symptom improvement, create a sense of being continuously supported and encouraged, and remind the user that they are actively taking steps to gain control over their symptoms. Finally, the alert-based functions, such as the “snippets”, may provide a means of buffering against or breaking the negative feedback cycles that contribute to the maintenance of many psychiatric conditions. For example, cognitive models of depression posit that maladaptive responses to negative automatic thoughts (such as rumination and decreased engagement in pleasurable activities) are a primary factor that maintains perceptions of low self-worth and associated depressive symptomatology [34]. Therefore, receipt of motivational messages or normalizing facts may be a means of ensuring that users receive some form of positive reinforcement each day, thereby buffering against the development of a negative cycle.

Interestingly, neither initiation nor completion of the psychotherapy modules was associated with treatment outcome. This finding was unexpected; as we had hypothesized that the core skills taught in the modules would be key to producing symptom reduction. The lack of association may be due to limited variability in use of the modules, as the overall rate of module completion was low. However, ours is not the first study to find a lack of association between module completion and treatment outcome. In a study examining the association between usage metrics and treatment outcome in an RCT of an Internet depression treatment program, Donkin et al [12] found that the proportion of modules completed was not associated with treatment outcome. The authors made two suggestions that warrant further investigation: (1) either the number of modules completed may be a poor indicator of benefit obtained, or (2) that the relationship between module completion and treatment outcome may not be entirely linear, as was originally thought. Indeed, it is possible that rates of module use only within a specific period of the intervention (ie, in the first few weeks) may be more strongly associated with therapeutic gain than total module completion rate. To address this possibility, future studies may wish to examine associations between treatment outcome and feature utilization within discrete stages of the intervention. Another possibility is that user-specific factors, such as the degree of therapeutic alliance with the program, may contribute more strongly to symptom improvement than the actual skills taught in the modules. To investigate this, we have administered self-report questionnaires of therapeutic alliance and have conducted a series of qualitative interviews to determine the degree to which users developed a rapport with the program (paper submitted for publication). Examining reasons for low module use from users who had especially low rates of module completion, or those who did not use the modules at all, will provide further information.

### Strengths, Limitations, and Future Directions

The current study examined whether specific patterns of program usage were associated with superior treatment outcomes in an RCT of myCompass, a mobile phone and Web-based self-help intervention for individuals with mild-to-moderate depression, anxiety, and stress. The current findings contribute to our understanding of the mechanisms through which eMental Health interventions exert their therapeutic benefits, by isolating the program features most strongly associated with improvements in symptoms and functional impairment.

Although our findings suggest that alerts are associated with both increased program engagement and greater treatment outcome, this is only a first step, and further research is needed to confirm our findings. Specifically, the current analysis used a correlational design in the single group of individuals who were assigned to receive the active myCompass intervention within the broader RCT. Future studies using random assignment to specific program features is needed in order to reach firm conclusions about the causal relationship between patterns of program usage and reductions in symptoms.

Along similar lines, better information about potential nonlinear relationships between patterns of program use and treatment outcome could be obtained by assessing symptom improvement at more frequent intervals. Assessing symptomatology at each week of the intervention may provide a greater insight into the usage patterns and points of disengagement of those with low rates of adherence, or alternatively, those who may be classified as “e-attainers”. This information could then be used to further develop and refine the features that show the strongest associations with symptom reduction, as well as to incorporate prompts at key junctures to assist in reducing rates of program attrition.

In the current study, the rate of module completion was approximately one quarter to one sixth of that expected, which is a limitation, and this may be partly due to the modules being unavailable via mobile phone. However, a lower-than-expected rate of module completion is not an issue that is unique to our intervention. Indeed, many self-paced eMental Health interventions have low rates of module completion, however, there is evidence to suggest that this may not necessarily equate to receiving a lower “dose” of the program. Studies suggest that individuals may derive substantial benefit from symptom tracking alone [35], and it may be that many users prefer this to completion of more in-depth modules. This again points to a need for further research into the most potent components of Internet interventions. If modules turn out to produce the greatest therapeutic benefit, then programs may benefit from evaluating methods of incorporating incentives that drive up rates of module completion. However, if the modules are associated with only marginal improvement compared to other features, it may be more beneficial to consider ways to maximize the potency of other features (eg, using reminders to prompt practice of therapy-based skills).

Finally, the sample used in the current study was predominantly female (160/231, 69.3%), university-educated (135/231, 58.4%), and around 40 years of age. Although sex, education, and age were not found to be strong predictors of program use, it is possible that rates of program engagement, or use of certain features, may differ in other populations. It is possible that rates of program usage may be lower in individuals with poorer literacy, or indeed, those who present with more severe forms of psychopathology.

### Conclusions

The current study examined associations between patterns of program usage and symptom improvement in myCompass, a Web and mobile phone-based intervention for mild-to-moderate depression, anxiety, and stress. The alert features of myCompass were most closely associated with symptom improvement, indicating that brief cues that signal self-monitoring or that provide positive reinforcement may be an especially potent feature of eMental Health interventions. Although data suggested a stronger association between symptom improvement and alerts received via email than via SMS, there was substantial overlap between use of email and SMS reminders. As such, future studies using random assignment to specific program platforms are needed to determine the most therapeutically beneficial platform for delivery of this program component.

## Abbreviations

DASS: Depression, Anxiety, and Stress Scales
EMA: ecological momentary assessment
RCT: randomized controlled trial
SMS: short message service
WSAS: Work and Social Adjustment Scale
